# How Can Music Engagement Address Loneliness? A Qualitative Study and Thematic Framework in the Context of Australia’s COVID-19 Pandemic Lockdowns

**DOI:** 10.3390/ijerph20010025

**Published:** 2022-12-20

**Authors:** Frederic Kiernan, Jane W. Davidson

**Affiliations:** Faculty of Fine Arts and Music, The University of Melbourne, Parkville, VIC 3010, Australia

**Keywords:** music engagement, loneliness, social isolation, COVID-19, perceived control, social connection, identity, mobility, wellbeing

## Abstract

Social isolation and loneliness are serious public health concerns. Music engagement can strengthen social connections and reduce loneliness in some contexts, although how this occurs is not well understood; research suggests that music’s capacity to manipulate perceptions of time and space is relevant. This study adopted a qualitative perspective to examine how music engagement shaped the experiences of residents of Victoria, Australia, during conditions of restricted social contact during the lockdowns of 2020. Semi-structured interviews explored participants’ lived musical experiences while giving focus to perceptions of time and space (e.g., how music helped restructure home and workspaces in response to lockdown regulations, or punctuate time where older routines were no longer viable). Interpretative Phenomenological Analysis of the interview transcripts identified five themes representing the key findings: (1) a super-ordinate theme of perceived control, which comprises four themes: (2) dynamic connection; (3) identity; (4) mobility; (5) presence. Each theme describes one generalised aspect of the way music engagement shaped participants’ perceptions of time and space during lockdown and supported their processes of adaptation to and coping with increased social isolation. The authors argue that these findings may inform the way music can be used to address loneliness in everyday life.

## 1. Introduction

The COVID-19 pandemic has not only had far-reaching impacts on human physical health, but has increased social isolation due to the physical distancing policies introduced to control the disease, which have drawn further attention to loneliness and social isolation as public mental health issues [1]. Social isolation arises when an individual’s social network is small and/or they have little social contact, while loneliness arises when an individual perceives themselves to be socially isolated. Whereas social isolation can be objectively quantified [2], loneliness is typically understood as a subjective feeling [3]. Social isolation and loneliness are elements of social connection, a multi-factorial construct that includes structural, functional and qualitative aspects of social relationships [4]. Mounting evidence shows that social isolation and loneliness can have serious consequences for physical health [5,6,7], with each having been found to predict premature mortality, depression, cardiovascular disease and cognitive decline [8,9]. This has led to calls for social connection to be advanced as a public health priority in societies where social isolation and loneliness are prevalent [4].

The COVID-19 pandemic and its associated physical distancing restrictions and stay-at-home orders revealed a need to appreciate the importance of addressing social isolation and loneliness as psychological and public health concerns [1]. Australia’s approach to controlling the spread of the virus during 2020 was relatively successful by international standards [10,11,12,13], with the impact being concentrated primarily in the state of Victoria. This relative success was largely the result of two extended periods of lockdown [14], the first of which was enforced nationwide for a duration of eight weeks from late March 2020, while the second was enforced in Victoria from early July in response to a second wave of infection. After almost four months of lockdown, then reported to be one of the “toughest in the world” [15], residents of metropolitan Melbourne could leave home for usual daily activities and retail businesses reopened [16]. By mid-February 2021, 70% of Australia’s recorded COVID-19 cases and 90% of Australia’s COVID-19 deaths had occurred in Victoria [17]. Despite the relative success of these lockdowns in curbing the spread of the virus, their impact on the mental health and wellbeing of Australians, including their experiences of social isolation and loneliness, was significant [1,10,18].

Approaches to tackling the problems of social isolation and loneliness have varied. Masi et al.’s [19] quantitative review of interventions identified four primary strategies typically used in programs aimed at reducing loneliness: (a) improving social skills; (b) enhancing social support; (c) increasing opportunities for social contact; and (d) addressing maladaptive social cognition. The researchers found that those addressing maladaptive social cognition were associated with the largest effect size. This finding indicated that some approaches may be more suited to addressing social isolation (e.g., by increasing social contact) rather than loneliness (e.g., a perception that requires subjective experience to be understood and managed). It is also consistent with Cacioppo and Hawkley’s [20] model of loneliness as a regulatory loop, whereupon lonely individuals tend to display sensitivities, expectations and behaviours (e.g., hypervigilance to social threats, negative social expectations, withdrawal) which have short-term self-protective features, but which may paradoxically reinforce loneliness over the longer term and lead to adverse health outcomes. This also suggests that interventions to reduce loneliness could be better targeted, for example, through cognitive behavioural therapies addressing empathy, or the identification of automatic negative thoughts about others and their reframing as possibly faulty hypotheses needing verification [19].

However, given the multi-factorial nature of social connection, and the need to understand influences and conduct analyses at micro and macro levels (e.g., genetic markers of susceptibility, cultural norms), various dimensions or types of loneliness have been proposed. Cacioppo et al. [21] differentiate “intimate loneliness” (the perceived absence of a significant other) from “relational loneliness” (the perceived absence of quality friendships or family connections) and “collective loneliness” (the perceived absence of an active network of social identities). These, in turn, map onto the three dimensions surrounding one’s attentional space as articulated in Hall’s [22,23] research on proxemics: “intimate space” (the closest space surrounding a person), “social space” (the space in which people feel comfortable interacting with family and acquaintances), and “public space” (a more anonymous space) [21]. Along with these spatial aspects, loneliness can also be understood as having temporal aspects, for example, in the unfolding of the experience of loneliness over time as well as its temporal position as a potential antecedent to emerging mental health symptoms [24]. This research therefore suggests that perceptions of time and space may play a role in the ways conditions of social isolation are perceived (i.e., the way loneliness is experienced), and that media capable of manipulating perceptions of time and space may in turn be capable of reducing feelings of loneliness among socially isolated people.

Individuals often use music to navigate experiences of distress or loneliness [25], although the way music offers support in such situations is theoretically contested and explained differently across disciplines. For example, studies in music psychology have shown that music can help listeners feel as though they are in the company of a comforting friend [26,27], feel understood and emotionally supported [28] and feel less alone [29], with the related benefits often explained in terms of social functions and psychological mechanisms [30,31,32]. Studies in community music therapy and music sociology have tended to focus on the ways music cultivates a sense of self and belonging by providing a medium for social agency and social action [33,34,35,36]. Such studies have highlighted that music is always “music plus” (i.e., music plus people and things in action in context), and that music can reframe identities, provide pretexts for social relating and provide metaphors and subject matter for personal and group-historical narratives, among other things [37]. Studies on the impact of music engagement and participation on loneliness have given focus to older people in residential care settings and/or their caregivers [38,39,40], a demographic that has stereotypically been associated with loneliness [41], although recent research has found that younger people may be at higher risk [42]. Given that loneliness and social isolation are serious public health concerns, further research within and across disciplines is needed to examine how music may address loneliness and support people who are socially isolated.

The role of music in responding to the COVID-19 pandemic has attracted attention from scholars, with a recent special issue of *Frontiers in Psychology* capturing 44 studies on the topic of “Social Convergence in Times of Spatial Distancing: The Role of Music During the COVID-19 Pandemic” (see [43]). Empirical–quantitative studies prevail in this literature, and have shown that music participation and engagement have supported the mental health and wellbeing of individuals in a range of contexts [44,45,46]. During the pandemic, music has helped people to relax, escape, lift their mood or keep them company [47], and has functioned socially as a mode of communication that can foster a sense of belonging [48]. One study found that Australians ranked music listening as the most effective artistic creative activity for making them “feel better” during the pandemic [44], while another found that young Australians (aged 18–24) during lockdown were more likely to integrate music into daily life, use music for emotion regulation, respond to music in embodied ways and use music to perform a social identity than participants in all older age groups [49]. Another large survey of over 5000 people with representative samples from three continents found that people experiencing increased positive emotions used music for solitary emotional regulation, while those experiencing increased negative emotions used music as a proxy for social interaction [50], highlighting the widespread importance of social connection as an aspect of musical responses to the pandemic.

A recent review of the research literature on music listening and music making during pandemic lockdowns also showed that music became a compensatory source of hedonic pleasure while fostering eudaimonic processes of meaning making, and has framed these wellbeing-related [51] insights explicitly in terms of social isolation [52]. Yet, to the best of our knowledge, the ways music helped shape peoples’ perceptions and experiences of time and space during pandemic lockdown has not been investigated. The COVID-19 pandemic thus generated social pressures that invite analysis of how music may have transformed perceptions and experiences of time and space during lockdown and, in so doing, acted as a technology for facilitating adaptation to and coping with conditions of increased social isolation.

Scholars across diverse disciplines and fields have long agreed that music can act as a medium for transforming experiences of everyday life, including music sociology [53], musicology [54,55], music therapy [56], cognitive science [57] and urban planning [58]. Insofar as this pertains to the health- and wellbeing-related outcomes of musical activity, Tia DeNora’s [34] grounded theoretical account of “music asylums” highlights that music’s capacity to transform perceptions and experiences of time and space is crucial, since music asylums can provide “respite from distress and a *place and time* in which it is possible to flourish” ([34], emphasis added). Two broad strategies contribute to creating such asylums, removal and refurnishing, where the former generates a sense of distance from one’s environment and helps to recover a sense of personal time and rhythm, while the latter involves creative, imaginative play, thus helping to remake or renegotiate social worlds [34]. Both strategies seek to achieve the same end: “Room or respite from irritant features of the environment, ontological security, control and creativity, pleasure, validation of self, sense of fit, flow, comfort, ease and a feeling of being in focus” [[34], p. 55]. This focus on practices lends itself to in situ ethnographic inquiry [34,53], which was made more difficult by the pandemic [59]. However, other qualitative approaches such as in-depth interviews can still provide insight into how music asylums are experienced, even if those experiences are understood in terms of generalising psychological themes and by proxy via retrospective, subjective accounts that do not capture the full complexity of real-time musical (inter)actions and the micro, often tacit, practices that render and reveal music as meaningful in non-verbal ways [60].

Recent research in cognitive science, especially that using 4E approaches, has supported many of DeNora’s claims, albeit from a different theoretical context. The 4E perspective holds that the mind is (1) extended, in that it is configured through interaction with tools outside the body [61]; (2) embodied, in that our whole body constitutes mental life and facilitates the main cognitive domains [62,63]; (3) enactive, in that cognition is a continuous activity regulated by processes of autopoiesis that precede reflection or abstraction [64]; and (4) embedded, in that bodily and environmental restrictions determine the behavioural organisation of living organisms and thus help to constitute cognitive processes [65]. The 4E perspective in cognitive science thus proposes that we offload thinking processes onto our bodies and the world. Insofar as this pertains to music, scholars such as Joel Krueger have echoed DeNora in arguing that music can provide a form of “affective scaffolding” by acting as a resource that facilitates this offloading ([66], see also [67]). As Krueger argues, music can “scaffold access to new forms of thought, experience and behaviour” and can thus be viewed as an aesthetic technology for “worldmaking” [66]. This is exemplified in the ways music can be used to construct and organise spaces and environments that can be inhabited, explored and manipulated [68]. For Krueger, this occurs via two distinct but interacting forms of spatial content that he proposes are intrinsic to musical consciousness: (1) music’s *locational* spatial content, which is the hearing of music as coming from somewhere in “visible space” (e.g., from “over there”, from the television, from behind me, etc.), and (2) music’s inner *structural* space, which is the perception of music as itself being a structurally organised soundworld with its own internal spatial properties (e.g., slow rhythms and sparse textures may help to generate a sense of vast space *in* the music) [66]. This intersection of psychology, music sociology and cognitive science provides a useful theoretical context in which to explore the potential roles of music in adapting to and coping with conditions of increased social isolation during the COVID-19 pandemic.

The aim of this study was to examine the experiences of Australians who used music to adapt to and cope with conditions of increased social isolation during pandemic lockdown. Consistent with qualitative semi-structured interviewing [69], our aim was to understand participants’ lived musical experiences, although, given the impact of the pandemic on participants’ regular routines and habits, the interviews tended to focus on issues related to time and space (for example, through discussions about how music helped facilitate the practical reorganisation of home and work spaces in accordance with lockdown rules, or how music could be used to punctuate or manipulate the perceived flow of time).

## 2. Materials and Methods

The University of Melbourne granted ethics approval to conduct in-depth, semi-structured interviews (ID 2056873.1). Participants were alerted to the study in two ways: (1) via an online survey, which included an invitation to participate in an interview [44], and (2) social media and university media announcements (e.g., Facebook posts, staff and student newsletters) specifically inviting participation in an interview. Eleven participants, all residents of Victoria, agreed to participate and were provided with a plain language statement and consent form (see Table 1). The first author conducted the interviews on Zoom over the period June–August 2020, when Australians had already endured the first nationwide lockdown and Victorians were just entering the second, longer lockdown. Participants were advised that the interviews were expected to take 30 min, although the actual length of interviews varied between 20 and 150 min depending on the participant. All participants were eligible to win a gift card worth AUD 200, and at the conclusion of data collection, the winner was randomly drawn and provided with their gift card. The interviews were audio-recorded and fully transcribed for analysis, with interviewees being given the option of using a pseudonym to protect their identity.

During the interviews, participants were asked to discuss the impact of the pandemic on their living and working arrangements, as well as the creative activities they had been undertaking during lockdown, and why. For each activity discussed, participants were asked to elaborate on who else (if anybody) was involved, to describe the context in which it occurred and whether the pandemic had impacted the way they normally engage in the activity. They were also asked to describe the perceived benefits of doing the activity, how the activity makes them feel, and which activities they might wish to do more of. Musical activities were not strictly divided into making/listening categories but were instead broadly conceived as anything pertaining to a “musical event” [34]. Transcripts were then coded in NVivo(version 12, QSR International, Burlington, Mass., USA) using Interpretative Phenomenological Analysis (IPA) to distil broader themes; this included bracketing initial, general notes about each interview and line-by-line analysis of the entire interview transcript to identify points of descriptive, linguistic or conceptual interest [69]. The process was iterative and the emerging codes and themes were repeatedly reconsidered and reanalysed in a dialogue between the two authors. This is because IPA provides insight into how individuals make sense of their respective “lifeworlds” [69], and gives focus to the meanings participants ascribe to their experiences, and as such the method acknowledges and makes use of a double hermeneutic: the interviewer making sense of the interview participant making sense of their experience. This necessarily omits potentially relevant information, for example, forms of habituated, practical action that the participant may deem unworthy of mention but which an ethnographer observing an event in real time may find to be relevant. However, it does allow for a greater focus on language as a means by which subjective experiences (e.g., of musical asylum or loneliness) are rendered intelligible to the participant and is practically feasible in contexts where the physical presence of an ethnographer may not possible, such as during a pandemic lockdown. The analysis of the interviews was performed by the first author and carefully re-read and cross-checked by the second author. The second author cross-checked the analysis by examining raw data files, looking at analysis categories and data listed within each, and sought examples which supported and/or refuted the cases. This check of robustness of the thematic categories and examples used is consistent with the IPA approach [69]. Multiple and detailed discussions of the data (interviewee transcripts, theme derivation and presentation of these for this article) were undertaken together to develop a robust analysis. The sample size used also accords with the principle that small sample sizes—usually up to six participants, but sometimes more [69]—can support greater depth of analysis using IPA. Most participants were from professional backgrounds and/or worked in managerial occupations and were of middle to high socio-economic status; further research would thus need to address this bias.

## 3. Results

Two strikingly common experiences were that all 11 participants reported (unsurprisingly) a reduction in opportunities for social contact due to physical distancing restrictions, and that all felt their sense of time and space had been warped during lockdown. For example, Caroline stated, “Time is just like, oh… what happened to April, May? It’s weird… One day just rolls into another”, while for Donna, April 2020 was “the longest month ever”. All participants reported that the spatial arrangements of their lives at home had changed during lockdown, with many converting shared kitchen or living spaces into temporary workstations or making other rearrangements to the home environment; as Rafael stated, “I’m literally locked in with just about everything in my life all around me… it’s where I sleep, it’s where I work out, it’s where I relax, it’s where I study… Everything is here.” Participants also spoke of the psychological impact of these temporal and spatial changes at home. Andrew, for example, described a feeling of detachment from his physical surroundings that was exacerbated by working long hours on the computer, a surreal experience of being both “here” and “not here” at the same time. He stated, “Sometimes I feel like I’m here [in the house] without being here… The household is going on around me, but I’m not part of it.” David also said that the experience of lockdown had triggered anxious tendencies in a similar way to “having a scab… and it [lockdown] opens up the wound again.” All participants thus experienced lockdown as a period of adjustment requiring a sense of perceived control, and their interviews all centred on perceptions and experiences of time and space and music’s role in adapting to these new conditions.

**Figure 1:**
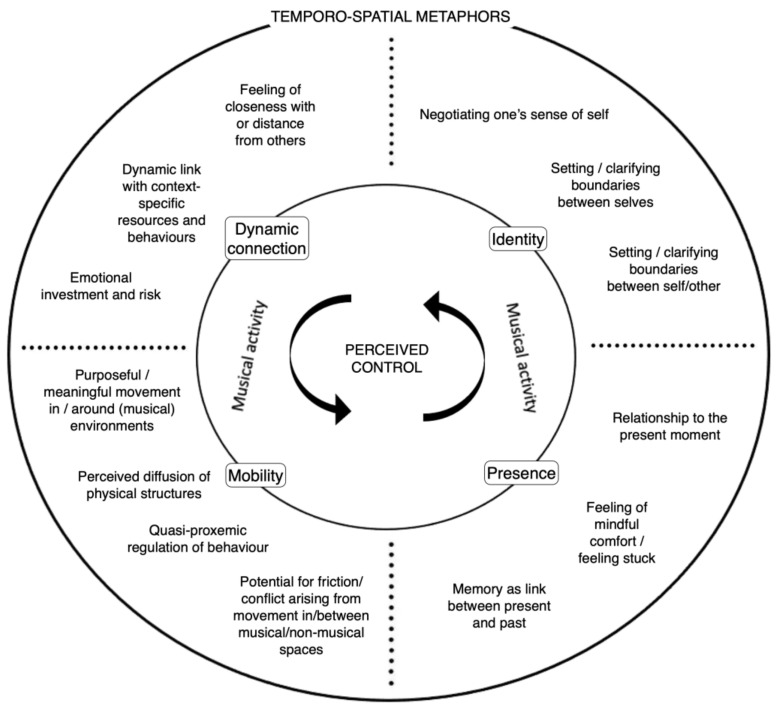
A thematic framework explaining music’s mediating role in adapting to and coping with conditions of social isolation during pandemic lockdown. The super-ordinate theme is represented at the centre; the four remaining themes are represented on the smaller, inner circle; the individual codes are represented in the largest, outermost circle. Musical activities shaped and were shaped by all aspects of the framework. This figure was developed by the authors for illustrative purposes.

The results of the analysis are discussed in terms of five key themes: (1) a super-ordinate theme of perceived control, comprising four themes: (2) dynamic connection; (3) identity; (4) mobility; and (5) presence. The inter-relationships between the codes, themes and super-ordinate theme in the resulting framework are outlined in Figure 1. Each of these themes describes one generalised aspect of the way music shaped participants’ perceptions and experiences of time and space and supported their processes of adaptation to and coping with conditions of increased social isolation during lockdown. It is important to note, however, that the inherent ambiguity and complexity of musical experiences means that these themes are not entirely mutually exclusive, and that some of the individual codes emerging from the analysis could easily have been applied to one or more themes (hence the use of dotted lines to loosely divide the codes into thematic groupings). The themes are discussed using indicative “gem” quotations that distil the essence of participant experience [70]. Some participants were more ready with fluent responses than others, so while all respondents discussed all five themes, some voices are slightly more present in this article than others due to the clarity of their reflections.

### 3.1. Perceived Control

The over-arching (super-ordinate) theme that emerged across all interviews was that of perceived control, a desire to feel as though one could assert and relinquish control over one’s life during lockdown as needed, and to use music for this purpose. This was expressed through, for example, discussions about controlling one’s experiences, emotions (emotion regulation), perceptions, environments, movements, attention and sense of self. Participants skilfully used music, consciously and unconsciously, to guide attention and scaffold emotion to achieve a range of outcomes, which are grouped together under four key themes to be discussed below (dynamic connection, identity, mobility, presence). However, some participants also discussed attempting to achieve a sense of perceived control through music explicitly and distinctly from these four themes (i.e., in a way that addressed the idea of having control over one’s life in general, rather than over some specific aspect of it). Participants also reported varying degrees of success in this regard, with some finding that music did not help; this super-ordinate theme therefore does not refer to the achievement of control, only the attempt to achieve it. Six gems from three participants (Devon, Bianca, Aaron) illustrate this use of music.

Devon, a university student, discussed the potential benefits and limitations of different aesthetic media in helping her achieve a sense of control over her life during lockdown. For example, her choice to engage in a specific creative or artistic activity depended partly on that medium’s affordances, and on the technical possibility of achieving a feeling of control over the medium itself. For Devon, music was a “messy” medium that lacked clear boundaries and parameters, and this influenced her engagement with music in this context. Devon said:

*Anything that includes messiness, I have not gone anywhere near [during lockdown]. Most of the creative things I’ve been doing are very controlled and ordered.*

*I haven’t been going that far into music or writing and things like that as much as I would before the pandemic. I think it’s definitely [about] control and order.*

*I’ve done more editing and design stuff. But I haven’t been doing… anything like that [music], [where] the parameters can be overwhelming.*

*When it’s creativity within a frame and within a task, I definitely found myself being drawn to that rather than being like, I can just do whatever.*

Music offered too many choices and possibilities for Devon. While certain creative activities did help her to build structure and achieve a sense of control in a context where previously effective strategies were no longer viable, this did not include musical activities.

For other participants, the choice to engage with a particular aesthetic medium was more strongly linked with external factors that were restricting the options available. For example, Bianca explained that her need to achieve a sense of control was partly due to her experience of coping with an abusive relationship and its breakdown during the pandemic, and she described how taking photographs on her iPhone while outside walking helped her through this experience, partly because her ex-partner did not have access to this device. Bianca’s photography had become a creative practice of resistance and an assertion of her agency—but music, unlike photography, did not provide Bianca with the same experience of control. She stated:

*I didn’t find a lot of relief in music [listening], which was interesting… I think a lot of the joy went, maybe. And maybe I just don’t find the songs and music that… I guess I don’t have control over it maybe… [Photography is] one way of just taking back some control, where everything is my interpretation.*

This comment also illustrates a broader trend in the findings, in which processes of achieving a sense of control over one’s life during lockdown were allied to processes of aesthetic interpretation (i.e., of finding “goodness” in music in whichever way). Moreover, these processes of aesthetic interpretation sometimes resolved controlling urges into feelings of acceptance of the difficult lockdown situation. For example, Aaron, a filmmaker, stated:

*[Music-making] definitely bonds us together; I have no doubt about that. One thing for me, it gave me… less performance anxiety. It was more important to have fun, and less important in what it sounded like and who gives a crap? It was more about having fun and accepting that we’re all in this lockdown period.*

Considerations of musical quality, of what makes music good or bad, were thus tied to the good work music did or failed to do in the lives of participants during lockdown. This provides an illustration of DeNora’s [34] grounded and contextual approach to understanding musical aesthetics, and while some participants found that music was not as helpful an aesthetic medium as others for adapting to life during lockdown, all reported attempting to use music to achieve a sense of perceived control over their lives in some way. The following four themes describe in greater detail the specific ways that participants did this, and temporospatial metaphors permeated these discussions.

### 3.2. Dynamic Connection

Participants used music to construct experiences of connection with other people (both co-habitants in lockdown as well as those in extended networks reached via digital means) that were dynamically linked with context-specific resources (e.g., instruments, music editing software, social media). These musical experiences were emotional and were coupled with reflections about participants’ own behaviour within and around the musical event or the observed behaviour of others. The emotional qualities of these experiences (e.g., intimacy, excitement, cold disinterest) were dependent on the affordances of particular resources and the ways resources were mobilised. This theme is thus discussed in terms of dynamic connection and is illustrated by six gem quotations from two contrasting examples (Jessica and Jennifer).

Jessica spoke about her most important musical experiences during lockdown in terms of an intense and joyful feeling of closeness with those around her. Jessica is a researcher who was spending lockdown at home with her husband and three teenage children, and she recounted how she and her family used shared music making and group improvisation sessions to practise listening skills that informed their social interactions and behaviour both within and outside of musical events. She said:

*If you’re the one starting off an improv… you need to set up a kind of a motif or a pattern that is reliable enough for everyone to find their place in it.*

*Music relates to other interactions with other human beings.*

*[Music improvisation] teaches you how to listen to other people before you jump in and how to have a conversation.*

*[Music] is pure joy… there’s no words really… everyone’s just listening to each other and finding ways to make this thing work and being playful and taking risks. But it doesn’t matter if someone does a weird note, because then sometimes we go, “Oh, let’s all go there”.*

Jessica’s comments illustrate how she conceived of social dynamics in terms of musical time and space (“before you jump in”, “let’s all go there”) and how musical sound could be sensitively manipulated in performance so that those in her musical (family) group could “find their place” in it. Through shared music making, Jessica observed that members of her household became more attuned to one another and thus more connected. By constructing temporary musical spaces that Jessica and her family could all occupy and manipulate through active participation, the social dynamics between family members in the home could be negotiated in the absence of other social outlets.

In contrast, Jennifer provided a somewhat less joyful account of producing an asynchronous choir performance using digital media as a substitute for live performance in accordance with lockdown restrictions. Jennifer also discussed this experience in terms of musical time-space and its emotional implications, especially regarding feelings of connection with other people. Jennifer is a professional university staff member involved in managing donor relationships, and was involved in a choir that would often perform live during important commemoration celebrations of national significance. However, Jennifer noted that, in order to comply with lockdown restrictions, musical arrangements had changed; each choir member now had to individually record a separate vocal track, and these were all subsequently compiled in an editing suite and broadcast on Australian television. A YouTube link was later shared with the choir members who watched it all separately in their homes. Of the experience, Jennifer said:

*It’s very different from the procedure of putting together a live concert where we rehearsed together, then we all get to experience the end result in the same instant, all standing next to each other… We were… listening to that from the “outside”, rather than at the moment it was being produced.*

*It was like a very, very delayed reaction to that performance… rather than actually the art of coming together and singing live.*

Jennifer’s comments reveal how changes in performance materials and the nature of choir participation during lockdown impacted the way in which the size of the musical event and the flow of time within it was perceived. This, in turn, led Jennifer to cautiously question the value of participating in the choir that way because of how it impacted a treasured feeling of connection with the other choir members. (Jennifer did not explicitly describe the event as negative but did repeatedly use a questioning tone when describing it as being “very different” from live performance.) Jennifer’s comments illustrate how the rewards of her choir participation arose in part from her emotional investment in an inherently dynamic musical medium (dynamic because of its ties to changing materials, settings and practices), and that this investment (i.e., caring “for” music) was somewhat risky. Jennifer spoke about the “art” of choir practice (of “coming together and singing live”) in a way that conveyed different dimensions of the aesthetic value of those experiences for her and which supported DeNora’s claim that musical aesthetics can be understood as grounded and contextual [34]. Nevertheless, Jennifer continued to investigate and use digital–technological alternatives as a proxy rather than give up entirely on attempting to bring people together in music. Both Jennifer and Jessica’s comments also illustrate how this theme of dynamic connection involved different types and degrees of risk; for example, Jessica perceived risky and playful musical behaviour as occurring within, and as a manipulation of, musical structure itself (e.g., the suggestion of a “weird note” by a band member), whereas Jennifer identified risk in the possibility that changes to choir practices resulting from lockdown restrictions might lead to an “art”, and the feeling of connection integral to it, being lost or compromised.

### 3.3. Identity

Participants spoke about how important music had become for negotiating one’s sense of self during lockdown in the absence of other social outlets, and how music helped them set or clarify boundaries between different versions of themselves (e.g., family roles or professional identities). These processes involved using music to think in temporospatial terms (e.g., “time/space for myself”) and to create and manipulate actual aesthetic spaces. This theme is considered in terms of identity because of its emphasis on music’s role in differentiating between current, and sometimes competing or contested, self-concepts or other identity-related constructs. Seven gems from three participants (Andrew, Chanda, Aaron) illustrate the theme.

Andrew, a university manager, spoke about how he was able to draw on memories of music making in the past to imaginatively and silently “hear” music in his head during lockdown. He also explained that his experiences of whistling or humming along to this music felt like he was “accompanying himself”, reflecting a shift in thinking facilitated by music in which he became both himself and his own accompanist. He said:

*I’m just imagining it [the music]… I’m just hearing it, I suppose, from my own memories [of performing].*

*I’ll start whistling along with what’s going on in my head… And I won’t really connect that I’m doing it… I’m not sort of actively doing it… it’s just sort of part of accompanying myself.*

*It’s a comfortable place… I probably default there a little bit, in the way that you can escape into a good book or into a good movie or whatever.*

Andrew’s comments illustrate that he experienced this silently “heard” music as having spatial dimensions that afforded a soothing escapism, but they also suggest that he understood this experience in terms of identity, although not explicitly. Andrew thus used music to open up an aesthetic space in which his self, and other possible selves, could be explored. This point was elaborated by other participants who spoke about the role of music in shaping and defining specific identities.

Chanda, an office worker, spoke about how music helped her create space between the different family roles and duties that her identity was bound to, and which she found particularly useful in the context of her crowded living situation. Chanda explained that her father’s disability meant he required constant care, and due to the increased perceived risk of COVID-19 infection through contact with his usual disability support workers, Chanda and her brother had taken over caring responsibilities completely, which, along with lockdown restrictions, had further restricted their opportunities for social contact. Chanda explained:

*The main thing is just having a space other than work and caring that I can be involved in… I think it’s definitely related to the living and work [situation], but also the caring roles as well… Music… it’s just really important, on a personal level.*

*[It’s] not physical space… I guess [it’s] kind of like a mental space. Because you’re still all in the home where everything blends together.*

Music thus allowed Chanda to create a sense of space between different “selves” associated with social and familial roles whose importance had become more pronounced during lockdown. In these musical spaces, other possible selves could emerge and be explored.

Similarly, Aaron’s discussion about his musical experiences during lockdown illustrate this theme further. In early 2020, Aaron sold his family home and he, his wife and two children all moved into a caravan and embarked on what was to be a year-long family trip around Australia. Before they arrived at the border of the neighbouring state, New South Wales, that border had closed. He and his family then had to move in with Aaron’s father-in-law, meaning that seven people were now living in a three-bedroom house. The caravan was now parked in the driveway, and this had become his office. Aaron spoke about how various creative media were associated with, and gave shape to, different personal and professional identities. These identities were serving different purposes during the pandemic, and they influenced how and when he would engage with those creative media. For example, he used the term “official creativity” to describe certain kinds of work that he had been doing during lockdown, which was linked with his professional identity and to the production of commercially viable products, as well as the ongoing administrative duties that came along with this work. “Unofficial creativity” was more ephemeral because he felt no enduring relationship with the artefacts it produced. Aaron thus engaged in different creative activities (including music making) in a manner that he identified as being either “for himself” (to have fun and support his mental health during lockdown), or otherwise, where “otherwise” included for his audiences as a professional filmmaker. He explained:

*I almost call it an official thing, because you make a film, but then it goes on for years because those films exist and there’s documents you’ve got to sign regarding it and all that kind of stuff.*

*The non-official creative output is more ephemeral, I just want to pick up the guitar and play, and I’ll put it down, and that bit’s done and dusted. So, I guess I would classify it in that way… That’s only for myself, not for anyone else… I don’t do them [“unofficial” creative activities] for any reason other than they’re fun, and mental health reasons.*

Music thus helped Aaron negotiate and strengthen his sense of personal identity during lockdown in relation to his professional creative identity as a filmmaker. This process was especially important to Aaron because of the pressure that the pandemic had added to his living and working situations. Music allowed him to scaffold and negotiate his sense of himself as a person with an identity distinct from his professional life.

### 3.4. Mobility

Participants discussed the importance of mobility in musical contexts, where mobility refers not to mechanical or accidental acts of movement across time and space, but rather movement infused with meaning and purpose [71]. Eleven gems from four participants (Rafael, Aaron, Jennifer, Bianca) show the range of ways it was articulated.

Postgraduate student Rafael had been confined to a small studio apartment while writing his PhD, and all shared spaces in his building (offices, outdoor terrace, etc.) had been closed; he was only able to leave for brief walks outside or to exercise in the stairwell. In this strictly isolated context, Rafael explained the importance of music thus:

*I tend to get quite immersed in the music, so I forget that I’m stuck in my room, sometimes a bit too much and I don’t do [my] work.*

*The creative activities for me, especially the music, is like, “Oh, here’s a new environment you can be in.” You know?*

*I’d say that’s the importance during the pandemic: it [music] allows for at least the mental teleportation to a different environment… It’s like geography. It’s like, “This is my own world now.”*

*It’s almost like it [music] diffuses the physical structures, in the sense that I’m aware of where I’m at, but somehow it extends. It’s more of an unaware thing. [It’s] because I’m focused on the music that the confined limitations of my space disappear, and when I don’t have the music, I’m very acutely aware of the structures around [me].*

Rafael’s purposeful use of music to manipulate his perception of surrounding physical structures served as an adaptive tactic, one which allowed him to exert agency by imaginatively moving through musical-structural space into new musical “environments”.

As noted above, Aaron was now living with six other people in a three-bedroom house, and working in a caravan. Aaron’s choices about what music to make, and how and where to make it, allowed him to generate temporary experiences of personal space through which he could address his occasional need to be alone and manage his relationships with others in a crowded home environment. Aaron said:

*I’ve just thought of this now… when I need my space inside… I usually play the electric guitar with headphones in… I don’t like talking to people when I’m engaged with the electric guitar.*

*When I’m playing the acoustic guitar, I’m often talking… I’m feeling happy and social and [people] talk to me.*

*If I’m playing in the caravan, it just means… they’ll hear me. They’ll know that dad’s finished work, and he’ll be in, in 10 min. Yeah, it’s semiotics. Sending them signals that I’ll be there shortly.*

*If I play inside, usually [my son] will pick up the trumpet and start playing something.*

Aaron’s comments highlight how he was able to use music to serve a quasi-proxemic function [72,73], by choosing specific instruments or performance locations in order to communicate messages about his need for personal space and how he wanted his co-habitants to behave. During lockdown, Aaron used music to invite interaction and discourage it by manipulating both music’s internal structural content (e.g., selecting specific instrumental timbres) as well as its locational spatial content (e.g., positioning musical sounds within physical environments).

The confinement resulting from pandemic lockdown also meant that music was sometimes experienced as an irritation or distraction by those unwillingly exposed to it, illustrating the potential for friction and conflict in this theme. For example, Jennifer described how her daughters responded to her piano practice:

*[They] have had to listen to me practise [the piano] every day and there’s no escape… They’ve started coming and asking me if I wouldn’t mind shutting the door.*

*They realise they can’t get away.*

Even though her daily piano practice had given Jennifer “a good mental relief”, it had also occasionally made relations in the house more tense. Bianca, an executive assistant, recounted a similar story, stating:

*I love music and I love singing, but… [I’ve] got a 17-year-old telling [me] to shut up.*

These comments highlight music’s role as a double-edged sword that can facilitate a sense of mobility during periods of enforced social isolation in some situations, while being a cause for conflict in others. Music offered the possibility of moving in and around alternative environments, and temporarily perceiving the diffusion of surrounding physical structures (e.g., the confining walls of a studio apartment). It allowed participants to demarcate temporary musical geographies of personal space which influenced the movements of co-habitants in crowded homes in a quasi-proxemic fashion. However, friction and conflict could arise from unwanted music listening situations from which there was no escape. More commonly, however, musical mobility infused meaning and purpose into the lives of participants, helping them to adapt to conditions of social isolation.

### 3.5. Presence

As the at-times chaotic emergency of the COVID-19 pandemic unfolded during 2020, participants used music to manipulate their experiences of time and space in a way that sometimes engendered feelings of being comfortably grounded in the present moment (in the “here and now”), but which could also lead to feelings of being stuck. Music functioned this way partly because of its links with memory and its strong capacity to induce memories [74,75]. As David, a bank manager, said, “when we hear music, we go, ‘Oh… we were out that night,’ or, ‘we were listening to that,’ and it brings a flood of those past emotions.” Moreover, memory induction can inspire actions in the present that can make the present more habitable. Accordingly, this discussion of presence explores how participants used music to manage their relationship with the present moment. The temporo-spatial aspects of musical experience helped participants adapt to conditions of pandemic social isolation by helping them to reposition themselves in time and at least attempt to recover a sense of personal time and rhythm in the “here-and-now”. This theme is illustrated by eight gems from three participants (Donna, Caroline, Andrew).

Donna, a digital marketer in financial services at a bank, spoke about the comfort that arose through using music to recall memories of simpler, pre-pandemic times while working from home. While her experiences could also be interpreted in terms of their spatial dimensions (e.g., she discussed how she used music to demarcate new working and living spaces in the home), of particular interest here is how she used music to recall memories which acted as a resource for facilitating change in the present, and how this helped her cope with the socially isolated conditions of life during lockdown. Donna said:

*We kind of turn [the radio] on in the morning and that’s in the kitchen all day… That’s comforting because that kind of takes me back to a time where that’s all you could do.*

*[In the past] you didn’t have music on your phone… [while also] looking at the television… the technology wasn’t there, so it’s kind of going back to a quieter time… it’s more mindful. It uplifts me.*

Donna intentionally used an older technology (the radio) to achieve a “quieter”, interpreted here as meaning less chaotic, experience. From a 4E perspective, this was both extended and embedded, in that the process was configured through specific external tools (the radio, but not the phone or television) and informed by environmental restrictions (social isolation due to lockdown). Music thus helped Donna recall memories of her past, but it also helped her alter her perception of time itself, whereupon her past and present became intertwined. Donna’s skilful use of radio music to overlay or inflect the present moment with elements from her past gave her comfort and relief from the anxieties of social isolation.

However, musical memories did not always lead to uplifting experiences for participants, and some struggled to achieve a feeling of being grounded in the present moment. Caroline, an academic, whose workload increased dramatically during the pandemic, spoke about how her guitar sat unplayed in her apartment, a reminder of how little she had progressed on the instrument since she began studying it many years ago:

*I just never got to that level of confidence [on the guitar].*

Because of the lockdown restrictions on social contact and movement, it was also more difficult for Caroline to be physically distanced from the instrument that was triggering these memories. It was a constant presence in her life that did nothing to ameliorate the stresses associated with isolation and increased workload:

*There’s been so many times when it’s like, oh, I have a guitar, I should actually pick that up and try it and then I felt like, no, I should probably work instead… It is that intermix of work and home.*

*I really should be playing that [guitar], I really should… I know it would be good for me… but maybe I’ll just go to sleep and start the whole exhaustion again tomorrow.*

For Caroline, musical memories could lead to feelings of being stuck in time and place, the unpleasant obverse of Donna’s mindful comfort. The current theme of presence is thus neither positively nor negatively framed and refers only to the fact that participants attempted to use music to grapple with and position themselves in the present moment. The idea of practising the guitar could induce feelings of exhaustion and disappointment for Caroline, linked with memories of past failures to achieve progress on the instrument, which in turn led to an unpleasant increase in pressure in the present.

As noted above, Andrew also described how he had been silently “playing” music in his head. He explained that this helped him to transition between “work mode” and “home mode” as a replacement for the commute between office and home that he was no longer making due to the pandemic. He said,

*I’m not sitting down and playing music… I’m listening to music in my head… in a somewhat vegetative state… I decompress.*

*[It’s] sort of a meditative kind of process… I find [it] particularly useful to move from work mode into a home mode when I don’t have a commute anymore.*

*The music I can just go to. It’s like I’ve downloaded it into my brain and it’s there when I need it.*

This inner “hearing” of musical memories allowed Andrew to inhabit and move around in musical-structural space in a way that helped him to refresh and reset his relationship with his surroundings, where, prior to the pandemic, it had been his surroundings that would change (in the commute from work to home). While this also obviously relates to the theme of mobility, these comments are discussed under the current theme because they further illustrate how music’s temporo-spatial aspects provided participants with room to at least attempt to feel more grounded in the present moment. Andrew used music to manage his own psychological state during the pandemic (to “decompress”), and although his body was sometimes conscripted into participating in this process (e.g., by whistling or humming along), Andrew’s account provided little evidence of “offloading” [66], suggesting that the extent to which cognitive processes are offloaded varies between people and contexts.

## 4. Discussion

This study aimed to examine the experiences of Australians who used music to adapt to and cope with conditions of increased social isolation during the lockdowns that occurred in 2020 in response to the COVID-19 pandemic. The interviews highlighted that the concepts of time and space were central to participants’ musical experiences during lockdown, and the Interpretative Phenomenological Analysis [69] of the transcripts identified five key themes—perceived control, dynamic connection, identity, mobility, presence—which each illustrate one generalised aspect of the way music was able to manipulate participants’ perceptions of time and space [34,66] and in turn shape their perceptions of social isolation itself. The findings are considered in terms of a theory of loneliness as perceived social isolation that is best addressed using strategies targeting maladaptive social cognition [19,20]. The literature documenting this approach to understanding loneliness has had relatively little to say about music as a mediator of perception and cognition and has tended to emphasise the potential impacts and benefits of cognitive behavioural therapies in “treating” loneliness by breaking the cycles of maladaptive social cognition and behaviour that can reinforce it [21]. However, we suggest that the current study provides novel insights into the potential roles music engagement can play in influencing perception and cognition [66] in conditions of social isolation and thus in reducing loneliness outside of clinical-therapeutic contexts, which can, in turn, advance the important cause of social connection and loneliness mitigation more broadly [2,4].

Specifically, the findings of this study showed that, in conditions of increased social isolation, participants used music to regain a sense of perceived control over their lives, and that musical activities helped achieve this by addressing four key needs: (1) a need for participants to feel connected with the people around them; (2) a need to negotiate and explore their identities, including through the clarification of boundaries between selves as well as between self/other; (3) a need to imaginatively move within, through and around (musical) environments and to regulate the movements of others in quasi-proxemic fashion; and (4) a need to manage their relationship with the present moment and to feel grounded in their conscious awareness of it.

The qualitative methodology of this study cannot determine which musical strategies that participants used to cope with social isolation were more effective than others, but this was not the aim of the study. In this respect, we recall DeNora’s [34] concern that:

*To think of music as something that can be administered like a dose of medication (whether a one-off or over time) so as to become better is, in short, perhaps as silly as asking which specific incident in my life over the previous 55 years or the previous 5 min led me to feel the way I do today as I enjoy smelling the roses in my garden.*

[p. 143]

Rather, we argue that the findings of this study illuminate a variety of roles that music has played in processes of adaptation to conditions of social isolation, and that these may provide a basis for further investigation of how music engagement may be purposively oriented towards addressing social isolation and loneliness more generally.

## 5. Conclusions

We conclude by suggesting that the themes emerging from this analysis provide a way of understanding how music can be drawn into frames of cultural activity along with other para-musical things (talking, people, settings, objects, etc.) such that music becomes a transformative force in an individual’s perception of social isolation, and, by extension, their experience of loneliness. These findings are timely given the growing recognition that social isolation and loneliness are serious public health concerns [1,4]. The themes of perceived control, dynamic connection, identity, mobility and presence help to illuminate how and why music can become important for people experiencing increased social isolation, and indeed these themes are sufficiently capacious that musical activity may be “dialled” towards one theme or another based on context-specific knowledge of an individual’s life circumstances and needs, without over-prescribing musical action. For example, an individual experiencing a major life transition, something known to potentially heighten feelings of loneliness [76], may benefit from engaging in musical activities geared towards exploring and strengthening their sense of self and their social identity. Similarly, just as mindfulness training has been shown to be helpful in reducing loneliness in randomised controlled trials [77], an individual struggling with feelings of loneliness in everyday life may find meditative musical activities, or the incorporation of music into the routine aspects of daily life (e.g., chores or daily tasks), to be of benefit. These aspects warrant much further consideration in future research. Moreover, following the COVID-19 lockdowns of 2020–2021, and as people return to work in various ways (e.g., full return to their office sites, partial work from home, full-time work from home), it is clear that music might have a strategic role in assisting people as they transition to different modes of working and relaxing in and around their work/home environments. Music’s potential to act as a resource for managing the “return to work” is certainly a topic that would merit further investigation. While this study could have benefitted from a more diverse group of interviewees, it nevertheless offers new insights into the ways music mediates perceptions of social isolation and provides a useful guide to the ways music engagement may be oriented towards addressing feelings of loneliness in everyday life.

## Figures and Tables

**Table 1:** Participants.

Name	Age Range	Gender
Aaron	45–54	Male
Andrew	45–54	Male
Bianca	45–54	Female
Caroline	35–44	Female
Chanda	18–24	Female
David	55–64	Male
Devon	18–24	Female
Donna	35–44	Female
Jennifer	45–54	Other/prefer not to say
Jessica	45–54	Female
Rafael	25–34	Male

## Data Availability

The data presented in this study cannot be shared or made publicly available for ethical reasons related to the privacy of the participants as outlined in the notice of ethics approval from the University of Melbourne (ID 2056873.1, 28 May 2020).
